# Age-standardized incidence and mortality rates of oral and pharyngeal cancer in Puerto Rico and among Non-Hispanics Whites, Non-Hispanic Blacks, and Hispanics in the USA

**DOI:** 10.1186/1471-2407-9-129

**Published:** 2009-04-28

**Authors:** Erick Suárez, William A Calo, Eduardo Y Hernández, Elba C Diaz, Nayda R Figueroa, Ana P Ortiz

**Affiliations:** 1 Department of Biostatistics and Epidemiology, School of Public Health, Medical Sciences Campus, University of Puerto Rico, San Juan, Puerto Rico, USA; 2 Puerto Rico Cancer Center, Medical Sciences Campus, University of Puerto Rico, San Juan, Puerto Rico, USA; 3 Cancer Control and Population Sciences Program, University of Puerto Rico Comprehensive Cancer Center, San Juan, Puerto Rico, USA; 4 School of Dental Medicine, Medical Sciences Campus, University of Puerto Rico, San Juan, Puerto Rico, USA; 5 Puerto Rico Central Cancer Registry, University of Puerto Rico Comprehensive Cancer Center, San Juan, Puerto Rico, USA

## Abstract

**Background:**

In the American region, Puerto Rico (PR) has the highest incidence of oral and pharyngeal cancer (OPC), but racial/ethnic differences have never been assessed and compared with other groups in the United States of America (USA). We compared the age-adjusted incidence and mortality rates of OPC between PR and among USA Hispanics (USH), Non-Hispanic Whites (NHW), and Non-Hispanic Blacks (NHB) to assess the burden of this cancer in PR.

**Methods:**

Analysis of the age-standardized rates (per 100,000) was performed using the direct method with the world standard population (ASR(World)) from 1998–2002. Annual percent change (APC) and Relative Risks (RR) were calculated using the Poisson regression model.

**Results:**

The incidence ASR(World) for men in PR was constant (APC ≈ 0.0%), in contrast, a decrease was observed among NHW, NHB, and USH men, although only USH showed statistical significance (APC = -4.9%, p < 0.05). In women, the highest increase in incidence (APC = 5.3%) and the lowest decrease in mortality (APC = -1.4%) was observed in PR. The ratio of the ASR(World) showed that in all racial/ethnic groups, men had approximately 2–4 fold increased incidence and mortality risk of OPC than women (p < 0.05). Men in PR had a higher mortality risk (p < 0.05) of OPC as compared to USH, NHW, and NHB; but among women, PR showed a significant excess of mortality only as compared to USH (est. SRR = 1.82, 95% CI = 1.41, 2.33).

**Conclusion:**

The overall higher incidence of OPC in men in PR as compared to USH, NHB, and NHW could be explained by the effect of gene-environment interactions. Meanwhile, the higher mortality from OPC in PR suggests limitations in the health-care access within this population. Further research is warranted to elucidate these findings.

## Background

Cancer remains one of the leading causes of morbidity and mortality worldwide [1], being a major public health problem in both industrialized and developing countries [2]. In 2002, an estimated 274,000 cases of oral and pharyngeal cancer (OPC) occurred worldwide, with almost two-thirds occurring in men [3]. Differences in the patterns of OPC occurrence worldwide reflect variations in the prevalence of specific risk factors within the regions with the highest incidence rates, such as tobacco and alcohol consumption in Western Europe, chewing of betel quid in South-Central Asia and Melanesia, and solar irradiation in the South Pacific [4,5].

In the American region, the age-standardized incidence rate (World) of OPC in 2002 showed different patterns of disease occurrence by sex and among countries. Overall, men showed higher incidence rates than women [5]. In South and North America, the highest incidences among men were observed in Brazil (8.3 per 100,000 men) and the United States of America (USA) (7.9 per 100,000 men), respectively [5]. In the Caribbean, Puerto Rican men showed the highest incidence (10.6 per 100,000 men) followed by Cuba (6.4 per 100,000 men) [5]. Nonetheless, the incidence of OPC in women in Puerto Rico (PR) was lower (2.5 per 100,000 women) when compared with other Western Hemisphere countries, such as the USA (3.4 per 100,000 women) and Cuba (2.8 per 100,000 women) [5].

Racial and ethnic differences in OPC incidence and mortality rates in the USA have been documented among USA Hispanics (USH), Non Hispanic Whites (NHW), and Non Hispanic Blacks (NHB), but not extensively characterized for Puerto Ricans living in PR [6,7]. Ethnic variations in the burden of OPC in the USA may be explained by differences in cultural and regional characteristics, health-related habits, or genetic constitution among these groups [8]. Other factors that perhaps contribute to this phenomenon include differences in access to screening and access to timely state-of-the-art treatments [9].

Studying the burden of OPC in PR is of special relevance as this is the fifth most frequent cancer type among men and the sixth most common cause of mortality in this group [10]. Although a previous study compared OPC mortality among Puerto Ricans and NHW in the USA for the 1973–1977 period [11], this study did not compare the Puerto Rican population with other racial/ethnics groups in the USA, such as USH and NHB. The comparison of PR's cancer statistics with that of other racial/ethnic groups in the USA is of interest, given the political and sociocultural relationships of PR with the USA [12], and given that the Puerto Rican population (similar to other Hispanic subgroups) [13] resulted from the admixing of the genomes of Spaniards (Europeans), Africans, and Taínos (Caribbean natives) [14]. Thus, a comparison of OPC incidence and mortality rates in PR with that of other racial/ethnic groups in the USA is not only necessary to understand differences and similarities in the occurrence of this cancer across these populations, but as suggested in the field of genetic epidemiology [15,16] it could also provide relevant information on the influence of genetic and environmental factors on disease occurrence. As a consequence, the main aim of our study was to assess the age-standardized incidence and mortality rates of OPC in PR as compared to USH, NHW, and NHB for the period of 1998–2002.

## Methods

### Data sources

Data sources for this analysis included the SEER program and the PRCCR. The SEER program is a cancer surveillance database that collects and reports incidence data from a sample of the USA population. The PRCCR has collected information on cancer in PR since 1951, being one of the oldest population-based cancer registries in the American Continent; The PRCCR is part of the National Program of Cancer Registries (NPCR) administered by the Centers of Disease Control and Prevention (CDC). The PRCCR uses the coding standards of the SEER and of the North American Association of Central Cancer Registries (NAACCR); thus the registry is fully comparable with SEER data. In the year 2003, a CDC audit concluded that 95.3% of all cancer cases diagnosed or treated in hospital facilities in Puerto Rico were appropriately reported to the PRCCR; a result comparable to the US median (95%) [17].

The third revision of the International Classification of Diseases for Oncology (ICD-O-3) was used to select all cases diagnosed from OPC from 2001 and later (site codes C000–C148) for this analysis [18]. Cases from 1998 to 2000, which were originally reported using ICD-O-2, were converted to ICD-O-3. OPC incidence statistics from 1998 to 2002 for PR were obtained from the PRCCR. Incident cases for the same time period for NHW, NHB, and USH in the USA were obtained with the SEER*Stat 6.3.5 software (National Cancer Institute Surveillance Program, Bethesda, MD) and based on SEER 13. The SEER program identifies Hispanic ethnicity by a combination of medical record review and matching surnames against a list of Hispanic surnames. The term Hispanic used throughout this study does not account racial differences within the US Hispanic population.

The International Classification of Diseases, Tenth Revision (ICD-10) was used to select deaths of OPC for this analysis from 1999 and later (C00–C14) [19]. OPC deaths from 1998 were coded using ICD-9 (site codes 140–149). For PR, mortality information was obtained from the PRCCR as reported by death certificates enacted by the Puerto Rico Health Department. For the USA, OPC mortality information was obtained from the SEER program as reported by the National Center for Health Statistics (NCHS). USA mortality cases were obtained for all states except Connecticut, Maine, Maryland, Minnesota, New Hampshire, New York, North Dakota, Oklahoma, and Vermont because of the large number of individuals with unknown origin or ethnicity for several years. The "Hispanics Index" as developed by the National Cancer Institute was used to exclude these states, where mortality statistics for Hispanics were deemed unreliable.

### Statistical Analysis

For each racial/ethnic group, we applied the direct method to compute OPC age-standardized incidence and mortality rates (per 100,000 persons) during 1998 to 2002, using the World Standard Population [20]. These rates were identified by ASR(World), either for incidence and mortality, and were computed annually in a period of five years. To assess the trend of OPC risk during 1998 until 2002, the annual ASR(World) were calculated by sex, as follows:  where w_j _is the proportion of persons in the *j*-th age group of the standard population (World Population), d^k^_ij _is the number of cases (new cases or deaths) in the *j*-th age group for the *i*-th ethnic group in the *k*-th year, and n^k^_ij _is the population in the *j*-th age group of the *i*-th ethnic group in the *k*-th year. The annual percent change (APC) of the ASR(World) was estimated, overall and by sex, using the joinpoint regression model [21]. The Joinpoint Regression Program [22] was used for the APC estimation with the following parameters: 1) log transformation of the rate, 2) zero joinpoint model, 3) Poisson model using rate, 4) uncorrelated error model, and 5) Hudson's method.

To assess racial/ethnic group's differences, the ASRs (World) were grouped during the study period, as follows: . Then, the ratio of two standardized rates  between two different groups were estimated with 95% confidence intervals [23], to assess significant differences in OPC incidence and mortality rates between PR as compared to USH, NHB, and NHW. This ratio was denoted as Standardized Rate Ratio (SRR).

In addition, specific incidence and mortality rates for different age groups were computed during 1998–2002, by sex. Based on these rates, the relative risks (RR) were estimated with 95% confidence intervals to determine relative differences among the study groups by sex and five-year age groups using the Poisson regression model [24]. The reference racial/ethnic group in the age-specific RR estimation was PR. The statistical analysis was performed with the statistical package STATA 10.

## Results

### Trends of ASR (World)

**Figure 1:**
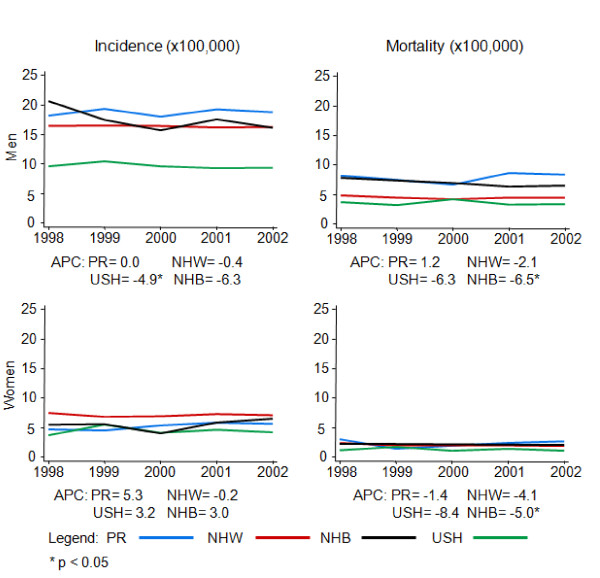
**Annual ASR (World) for oral and pharyngeal cancer incidence and mortality, 1998–2002**.

The annual ASR (World) for incidence of OPC during the study period (1998–2002) showed different patterns among the racial/ethnic groups studied (Figure 1). Men in PR showed a constant pattern for incidence from 1998 to 2002 (APC ≈ 0.0%), in contrast, a decrease was observed among NHW, NHB, and USH; although it was statistically significant only among USH (APC = -4.9%, p < 0.05). For mortality rates, declining trends were also observed for USH, NHW, and NHB men; although an increasing trend was observed among men in PR (APC = 1.2%). Among women, increasing incidence patterns were observed among PR (APC = 5.3%), NHB (APC = 3.0%), and USH (APC = 3.2%), but not for NHW (APC = -0.2). Meanwhile, although women in PR (APC = -1.4%) and in all other racial/ethnic groups showed declining mortality trends, this trend was only significant among NHB women (APC = -5.4%, p < 0.05).

### ASR (World)

The ASR(World) for 1998–2002 showed that in all racial/ethnic groups, men had approximately 2–4 fold increased incidence and mortality risk of OPC than women (p < 0.05). For example, men in PR were associated with 3.6-fold increased incidence (95%CI: 3.1, 4.1) and 3.5-fold increased mortality (95% CI = 2.78, 4.47) when compared to women. For the sex comparison in the other racial/ethnic groups, these fold increased risks were similar with those of NHB (Table 1). Among men, the ratio of the ASR(World) for incidence of OPC showed that PR had 1.96-fold increased risk (95%CI: 1.69, 2.29) compared to USH and 1.14 fold increased risk (95%CI: 1.05, 1.22) compared to NHW; no significant difference (p > 0.05) was seen between PR and NHB. For mortality, PR had a 1.15–2.27 fold increased risk (p < 0.05) from OPC compared to USH, NHW, and NHB. Among women, no significant differences (p > 0.05) in incidence rates were observed between PR and USH and NHB, although women in PR showed a 36% (est. SRR = 0.74, 95% CI = 0.65, 0.83) lower risk when compared to NHW. Meanwhile, women in PR showed a significant excess (p < 0.05) of mortality of OPC as compared to USH (est. SRR = 1.82, 95% CI = 1.41, 2.33), although no differences were observed between PR and NHW or NHB (P > 0.05).

**Table 1:** ASR(World) for incidence and mortality (per 100,000) for oral and pharyngeal cancer during 1998–2002.

	Age Standardized Rate (ASR)				Standardized Relative Ratio^a ^(SRR)		
	
	PR	USH	NHW	NHB	PR vs. USH^b^	PR vs. NHW^b^	PR vs. NHB^b^
**Incidence^c^**							
Male	18.5	9.5	16.3	17.3	1.96 (1.69, 2.22)	1.14 (1.05, 1.22)	1.08 (0.96, 1.19)
Female	5.2	4.3	7.1	5.4	1.20 (1.00, 1.45)	0.74 (0.65, 0.83)	0.95 (0.81, 1.12)
SRR Men vs. Women^b^	3.56 (3.10, 4.12)	2.20 (1.83, 2.65)	2.30 (2.21, 2.41)	3.17 (2.79, 3.45)			
**Mortality^c^**							
Male	7.9	3.5	4.4	6.8	2.27 (1.92, 2.70)	1.79 (1.59, 2.00)	1.15 (1.01, 1.30)
Female	2.3	1.2	2	2.1	1.82 (1.41, 2.33)	1.10 (0.88, 1.35)	1.09 (0.86, 1.33)
SRR Men vs. Women^b^	3.50 (2.78, 4.47)	2.80 (2.36, 3.35)	2.17 (2.10, 2.23)	3.29 (3.03, 3.46)			

^a ^The ratio of two ASR (World) with 95% confidence interval between parentheses.

^b ^Reference group

^c ^×100,000

### Relative Risks

**Figure 2:**
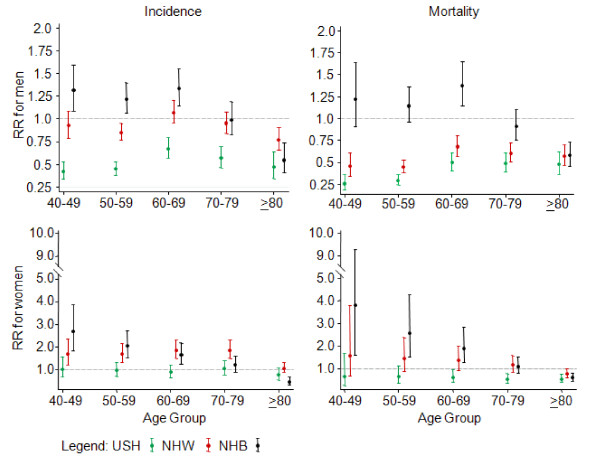
**Relative Risks' (95% CI) for incidence and mortality of oral and pharyngeal cancer in the Non-Hispanic Whites (NHW), Non-Hispanic Blacks (NHB), and the USA Hispanics (USH) when compared to Puerto Rico, 1998–2002**.

When the incidence and mortality of OPC in PR were compared with the other racial/ethnic groups for each specific sex and age-group, various patterns were observed during the period 1998–2002 (Figure 2). When compared to Puerto Rican men, their NHW, NHB, and USH counterparts in the continental USA had lower incidence in the age group = 80 years old (p < 0.05). In all other age groups, USH also had a lower incidence when compared with PR; whereas, NHB men showed higher incidence in the age groups 40–49, 50–59, and 60–69 (p < 0.05). Meanwhile, USH and NHW men showed lower mortality than men in PR in all age groups (p < 0.05); whereas, NHB had a significant higher risk of death (p < 0.05) at the age of 60–69 as compared to PR. Among women, NWH and NHB had a higher incidence (p < 0.05) of OPC when compared to women in PR, in the age groups 40–49, 50–59, and 60–69; whereas no age-group specific differences were seen between PR and USH (p > 0.05). Regarding mortality, only NHB women between 40 to 69 years of age had increased risk of death from OPC as compared to PR (p < 0.05); whereas, NHW and USH women had similar mortality in these age-groups.

## Discussion

During the study period, 1998–2002, the ASR(World) of OPC incidence and mortality were higher in men in PR than among USH, NHB, and NHW men. This higher burden of OPC in PR is consistent with studies performed in the 60's and 70's comparing cancer risk in PR and the USA [12,25]. Moreover, our study adds the comparison of these rates with two additional racial/ethnic groups (USH and NHB). Contrary to declining incidence and mortality trends observed for men in the other racial/ethnic groups, men in PR had a constant trend for incidence and increasing mortality trends. Although women in PR had lower incidence rates of OPC than NHB and NHW women, they had the highest increase for incidence and the lowest decrease for mortality when compared to the other three racial/ethnic groups. These results highlight a health disparity for PR that warrants further investigation.

The observed high burden of OPC in PR and increasing patterns of disease occurrence in this population are potentially the result of multiple factors, including lifestyle factors, genetics, acculturation, and screening practices within this population. Tobacco smoking explains some of the sex and racial differences in OPC risk [26]. A large case-control study of OPC in the USA showed that variation in the prevalence of use and the risks associated with tobacco and alcohol accounts for much of the higher incidence among men than women and among NHB as compared to NHW. [27]. In PR, it has been estimated that the attributable risk of OPC due to alcohol and tobacco use is around 76% (95%CI = 65–87%) for men and 52% (95%CI: 28–75%) for women [28]. Similar attributable risks have been reported in the USA [29].

Another study in PR also showed that OPC cancer in PR is strongly associated to liquor consumed straight, suggesting that alcohol concentration is a risk factor for OPC, independent of the total quantity of alcohol consumed [30]. In addition, risks associated with combined exposure to tobacco were also more pronounced when subjects drank liquor straight. In PR, straight liquor intake is reported more commonly among men (41%) than in their NHW counterparts in the USA (18%) [31]. Furthermore, locally produced, homemade rum is commonly consumed in PR and has been suspected to contain potentially carcinogenic contaminants [32]. Nevertheless, a study suggests that the risks associated with drinking homemade rum in this population are similar to those of other types of liquor [30].

Another factor that may explain the excess of OPC risk is the use of tobacco products. According to the Behavioral Risk factor Surveillance System (BRFSS), 14.5% of the adult population in PR reported cigarette smoking in 1996 and just 12.2% in 2007 [33]. Smaller studies conducted in PR prior to 1996 (1982 and 1989) suggest a declining trend in cigarette consumption. The prevalence of smoking has also consistently declined for the last three decades for NHW, USB, and USH [33,34]. Even with the declines reported for all the studied groups, PR has had a historically lower prevalence, almost one third, of cigarette smoking than NHW, NHB, and USH, which does not support the higher burden of OPC in our population. Nonetheless, this needs to be interpreted with caution as a delay of several decades is usually apparent between the first time of exposure to tobacco and the development of oral malignances [35]. In addition, given that historical data on tobacco consumption in PR is scarce, its difficult to correlate tobacco use and OPC risk in this population through the use of ecologic measures [36].

Genetic predispositions in PR should also be considered, as these predispose to the development of OPC at an early age [37]. In a population-based case-control study conducted in PR, the null GSTM1 genotype was associated with a marginally significant decrease in oral cancer risk (est. OR = 0.6, 95%CI: 0.3–1.0) [38]. The same study concluded that oral cancer risk augmented with increasing cigarette use among subjects with the GSTT1-present genotype (est. OR = 9.5, 95%CI: 3.0–30, among the heaviest cigarette users). Thus, further studies are warranted to determine if the frequency of these genetic polymorphisms, among other well known oncogenes, in PR is higher than among USH, NHW, or NHB; or if gene environment interactions vary among these groups.

Some attention also should be drawn to other lifestyle, immunologic, and environmental potential risk factors for OPC in PR, as these could be influencing the increased burden of OPC in this population. Relevant risk factors for OPC include mouthwash use [39], poor oral hygiene and poor dentition [40], oral cavity infections and diseases, denture sores, oral mucosal lesions [41], and sunlight exposure [42]. Also, exposure to sexually transmitted infections, such as the human papilloma viruses (HPV) [43], herpes viruses [37], and human immunodeficiency virus (HIV) [44] could impact OPC risk. Although no population-based data on the prevalence of HPV or herpes viruses has been published for PR that might explain the observed patterns of OPC occurrence, a higher incidence of HPV has been observed in developing countries of Latin America (33.5 per 100,000) and the Caribbean (33.5) [45] as compared to North America (< 15 per 100,000). Meanwhile, PR ranks fifth among all USA's states and territories in the rate of reported AIDS cases (26.4 per 100,000) [46]. Thus, a potentially high burden of these infections agents could be influencing the burden of OPC in PR, although additional population-based data is warranted.

In addition, dietary insufficiencies, specially fruit and vegetables, and low levels of serum nutrients such as carotenoids [47] could play a role in the observed results. Several studies have indicated a protective role for OPC of fruits and vegetables, although the specific agent is uncertain. For example, folates (found in a variety of foods, particularly green leafy vegetables, grain products, and orange juice) have long been hypothesized to be related to cancer risk [48]. A study conducted in PR showed that folate intake from fruit decreased OPC risk (p = 0.001) but other dietary folate sources showed no clear association (p > 0.05). In PR, fruit and vegetable consumption (5 or more per day) has decreased in the last decade (1996–2007) from 20.3% to 13.7%; meanwhile, the consumption for the USH, NHW, and NHB population has not showed a decrease and remains higher than that in PR [33]. This low consumption of fruit and vegetables as a direct source of folate may help to explain the higher incidence of OPC in PR. Nonetheless, historic nutritional information for PR is scare, limiting our ability to further explain the observed trends. Future studies should elucidate the role of diet on OPC risk PR.

The annual ASR (World) for mortality of OPC showed a decline in all racial/ethnic groups, except for men in PR. These declines could be explained by the participation in early detection programs, particularly of women [49-51]. This disparity for Puerto Rican men suggests that access to advances in treatment, screening, and early detection are not optimum in this population. In fact, a recent study by Morse found disparities in the detection of very early oral cancer in PR as compared with the USA [7]. Studies in this area are necessary to elucidate this argument. In addition, efforts must be made to increase the prevalence of OPC screening in PR, as early detection of OPC lesions is the most important factor in the prognosis and cure of this cancer.

Due to the acculturation process, it has been suggested that Hispanic migrants have cancer rates similar to NHW [3,8]. However, our results showed that USH men and women have lower incidence of OPC than NHW but a very similar mortality, a fact that has been explained by several factors, such as differences in health practices and health care access [3,8]. Among the Hispanic community living in the continental USA, acculturation occurs more markedly when they arrive to this country. Meanwhile, in the Hispanic population living in PR, acculturation has occurred through the close sociopolitical relationship with the USA since 1898 [12]. Given that the population of PR has experienced an acculturation process different from that of USH, the comparison of OPC incidence and mortality between these groups and the NHW and NHB populations is essential for further understanding differences and similarities of disease occurrence between these groups, that permit the identification of health disparities. However, because of the differences in the populations included under the broad heading of "United States Hispanics" [52] we cannot conclude that acculturation alone can explain the differences observed among Puerto Ricans and USH.

## Conclusion

In conclusion, our study shows an overall higher incidence of OPC in men in PR as compared to USH, NHB, and NHW. This result could be explained by the effect of gene-environment interactions. Meanwhile, the higher mortality from OPC in PR suggests limitations in the health-care access within this population. Further research is warranted to elucidate these findings. A comparison of OPC incidence and mortality between the population residing in PR and the Puerto Rican immigrants living in the continental USA would permit a more appropriate comparison that minimizes genetic differences, and contributes to the better identification of social, lifestyle, environmental, and biologic risk factors associated to disease occurrence and death in the Puerto Rican population.

## Competing interests

The authors declare that they have no competing interests.

## Authors' contributions

ES and APO participated in the acquisition of funding, designed and directed the study, supervised the data analysis, and wrote the manuscript. WAC participated in the acquisition of data, performed most of the data analysis, and took part in interpreting the results and writing the manuscript. EH helped in data acquisition and the statistical analysis. NRF worked in the data collection and data cleaning process of the data of PR and facilitated its acquisition. ECD provided consulting for oral and pharyngeal cancer. All authors reviewed and approved the final manuscript.

## Pre-publication history

The pre-publication history for this paper can be accessed here:

http://www.biomedcentral.com/1471-2407/9/129/prepub
